# Strengthening Arboviral Epidemic Response Through Entomological Surveillance: Insights from Bobo-Dioulasso, Burkina Faso

**DOI:** 10.3390/cimb48010078

**Published:** 2026-01-13

**Authors:** Zouéra Laouali, Hadidjata Kagoné, Thérèse Kagoné, Louis Robert Wendyam Belem, Hamadou Konaté, Ali Ouari, Alidou Zango, Saidou Ouedraogo, Raymond Karlhis Yao, Watton Rodrigue Diao, Olivier Manigart, Adoul-Salam Ouédraogo, Abdoulaye Diabaté, Olivier Gnankiné, Moussa Namountougou

**Affiliations:** 1 Centre MURAZ, Institut National de Santé Publique (INSP), Bobo-Dioulasso 01 BP 390, Burkina Faso; laoualiidi76@gmail.com (Z.L.); kagonehadi@gmail.com (H.K.); therese.kagone@gmail.com (T.K.); louisro2007@yahoo.fr (L.R.W.B.); hakonate89@gmail.com (H.K.); ouari_ali@yahoo.fr (A.O.); alidouzango01@gmail.com (A.Z.); saidoumuraz1@gmail.com (S.O.); karlhisyao@gmail.com (R.K.Y.); a-s.ouedraogo@yahoo.fr (A.-S.O.); npiediab@gmail.com (A.D.); 2 Unité de Formation et de Recherche en Sciences de la Vie et de la Terre, Centre d’Excellence, Africain, Université Nazi BONI, Bobo-Dioulasso 01 BP 1091, Burkina Faso; 3 Institut de Recherche en Sciences de la Santé (IRSS), Bobo-Dioulasso 01 BP 2779, Burkina Faso; 4 Centre des Opérations de Réponse aux Urgences Sanitaires (CORUS), Institut National de Santé Publique (INSP), Bobo-Dioulasso 01 BP 1500, Burkina Faso; rodriguediao@gmail.com; 5 Organisation Ouest Africain de la Santé (OOAS), Bobo-Dioulasso 01 BP 153, Burkina Faso; olivier.manigart@gfa-group.de; 6 Institut Supérieur des Sciences de la Santé, Université Nazi BONI, Bobo-Dioulasso 01 BP 1091, Burkina Faso; 7 Département de Biologie et de Physiologie Animales, Université Joseph KIZERBO, Ouagadougou 03 BP 7021, Burkina Faso; olignankine@gmail.com

**Keywords:** dengue, chikungunya, yellow fever, virus, detection, real-time PCR, mosquito, Bobo-Dioulasso

## Abstract

Arboviral diseases are emerging public health challenges in Burkina Faso, largely driven by the proliferation of *Aedes aegypti* mosquitoes in the environment. Effective surveillance of arbovirus circulation is critical to inform interventions. From August 2022 to June 2023, we implemented a comprehensive entomological surveillance platform in five sectors of Bobo-Dioulasso. Surveillance methods included oviposition traps to collect eggs, larval surveys in some concessions per sector conducted bimonthly, and adult mosquito collections using BG-Sentinel traps and Prokopack aspirators. Mosquito samples colonized by *Ae. aegypti* were identified morphologically, confirmed by conventional PCR, and screened by RT-PCR for dengue (DENV), chikungunya (CHIKV), yellow fever (YFV), and Zika (ZIKV) viruses. Molecular analysis detected dengue virus and yellow fever virus in mosquito pools from sector 22 and chikungunya virus in sectors 9 and 26; no Zika virus was found. This study demonstrates the successful establishment of an integrated entomological surveillance platform capable of capturing the spatial and temporal dynamics of arboviral vectors and virus circulation in Bobo-Dioulasso. The identification of active dengue and chikungunya transmission underlines the urgent need for sustained vector monitoring and targeted control strategies. Our approach provides a scalable model for arboviral disease surveillance and epidemic preparedness in West African urban settings.

## 1. Introduction

Arboviral diseases such as dengue, Zika, yellow fever, and chikungunya have recently undergone significant geographic expansion and resurgence of outbreaks, elevating them to global public health emergencies. Rapid urbanization and globalization through socio-economic, behavioral, and environmental factors, and case management are major factors of arbovirus emergence in urban settings worldwide [1,2,3]. The arbovirus (arthropod-borne virus) encompasses multiple viral families transmitted to vertebrates via bites from hematophagous arthropod vectors, including mosquitoes, ticks, and sandflies [4,5]. Dengue virus (DENV) transmission to humans is primarily mediated by mosquitoes of the Aedes genus [6,7]. Although dengue typically resolves spontaneously within days, severe forms with hemorrhagic manifestations occur, particularly in Southeast Asia and South America [8,9]. Among invasive mosquito species, *Aedes* is recognized as one of the most widespread globally, having colonized all five continents by exploiting human transportation networks [10,11]. This species adapts to temperate climates via diapause eggs, conferring cold resistance and facilitating survival during unfavorable conditions [12]. According to the World Health Organization (WHO), in 2018, approximately 3.9 billion people in 138 countries were at risk of dengue infection, with an estimated 390 million annual infections worldwide, including 96 million clinical cases, 500,000 hospitalizations for severe dengue, and 20,000 deaths, predominantly among children [13].

The year 2019 marked an unprecedented peak, with cases reported in 129 endemic countries [14]. In West Africa, *Aedes aegypti* remains the principal dengue vector [15,16]. As of 2023, half of the global population is considered at pandemic risk, with 25,000 deaths attributed to dengue. Between January and October 2023, 180,634 dengue cases (20,736 confirmed) and 462 deaths (case fatality rate 0.2%) were reported in 14 African Union member states [17,18,19]. Burkina Faso experienced its first recorded dengue epidemic in 1925 [20], with re-emergence from 2013 marked by annual outbreaks that have become a major public health issue. Vector control remains the cornerstone of dengue management but faces significant challenges due to increasing insecticide resistance among mosquito populations [21,22]. A severe dengue epidemic occurred in 2016, primarily in Ouagadougou, underscoring the urgent need for robust vector surveillance systems [23,24,25]. In response, Burkina Faso’s health authorities have prioritized establishing adaptive surveillance and outbreak management systems [19,26].

In the absence of vaccines or specific treatments, vector control targeting *Aedes* larvae and adults remains the main intervention. However, the emergence of insecticide resistance limits the efficacy of approved compounds. Two major outbreaks in 2016–2017 led to reinforcement of surveillance through sentinel sites in the Guiriko region [21,27]. Despite multiple efforts, dengue fever continues to rage. In 2023, Burkina Faso experienced a dengue epidemic, marked by a cumulative total of 154,867 suspected cases (diagnosed by syndromic diagnosis). Of these, 70,433 cases were probable (confirmed by rapid detection test), resulting in 709 recorded deaths [28]. The two epicenters were the most affected, particularly the Guiriko region. The clinical overlap of dengue, Zika, yellow fever, and chikungunya complicates accurate diagnosis, increasing the risk of misclassification. Since early 2023, chikungunya cases have also been reported in Burkina Faso and neighboring Senegal, with the first confirmed case notified in September 2023 in the Pouytenga district. By October 2023, 92 chikungunya cases were confirmed in Burkina Faso, prompting intensified surveillance and control measures [29,30]. Despite updated clinical management guidelines, morbidity and mortality remain significant, emphasizing the need for improved epidemiological and entomological data. Limited information exists on the bioecology of key arboviral vectors and the prevalence of arboviruses in Burkina Faso [31,32]. Given *Aedes aegypti* global dispersal, ecological adaptability, and capacity for insecticide resistance [33], understanding vector susceptibility profiles is essential for effective control. This mosquito thrives in urban and peri-urban environments by exploiting diverse artificial breeding sites, contributing to its high population densities in densely populated areas.

Therefore, surveillance systems integrating pupae and adult monitoring are critical for timely vector control. Current vector control tools are often costly, maintenance-intensive, and variably effective, underscoring the need for sustainable and innovative approaches that incorporate environmental considerations.

In a context marked by the emergence and increasing recurrence of arboviral epidemics, strengthening epidemic preparedness and response capacities depends heavily on the availability of reliable and up-to-date entomological data. In Bobo-Dioulasso, Burkina Faso, the establishment of an entomological surveillance system addresses a critical gap in the early detection of *Aedes* mosquitoes, the primary vectors of dengue and other arboviruses. This pilot initiative forms part of an integrated prevention strategy and provides timely information on vector presence, population dynamics, and ongoing arbovirus circulation. While comparable surveillance systems remain scarce in Burkina Faso and, more broadly, across Africa, existing studies often focus on operational aspects of health response mechanisms with limited integration of vector ecology. This study, therefore, seeks to illustrate, using Bobo-Dioulasso as a case study, how a systematic and real-time entomological approach can strengthen prevention, preparedness, and response to arboviral epidemics while more effectively guiding vector control interventions.

As this work represents a pilot study and given the large number of emerging viruses and the limited availability of costly laboratory resources, it was not feasible to target all arboviruses of public health interest. Therefore, we focused on the most prominent and recurrent epidemic arboviruses in the subregion. Our focus in this experiment was on arboviruses vectored by *Aedes* spp. (ZIKV, DENV, CHIKV, and YFV). The collection of *Culex* and *Anopheles* mosquitoes was incidental and indicates that *Aedes* mosquitoes may compete with these genera for ecological resources. To ensure an adequate probability of detecting arboviral infection, sampling was maximized by deploying diverse trap types in strategic locations, using species-specific attractants, and adhering strictly to trap maintenance protocols.

From August 2022 to June 2023, we implemented a comprehensive entomological surveillance platform across five sectors of Bobo-Dioulasso. Surveillance activities included ovitraps for egg collection, bimonthly larval surveys in selected concessions, and adult mosquito sampling using BG-Sentinel traps and Prokopack aspirators. Adult mosquitoes, predominantly *Aedes aegypti*, were identified morphologically and confirmed by conventional PCR, then screened by RT-PCR for dengue virus (DENV), chikungunya virus (CHIKV), yellow fever virus (YFV), and Zika virus (ZIKV). Molecular analyses detected DENV and YFV in mosquito pools from sector 22, and CHIKV in sectors 9 and 26; no ZIKV was identified.

## 2. Materials and Methods

### 2.1. Study Sites and Design

**Figure 1:**
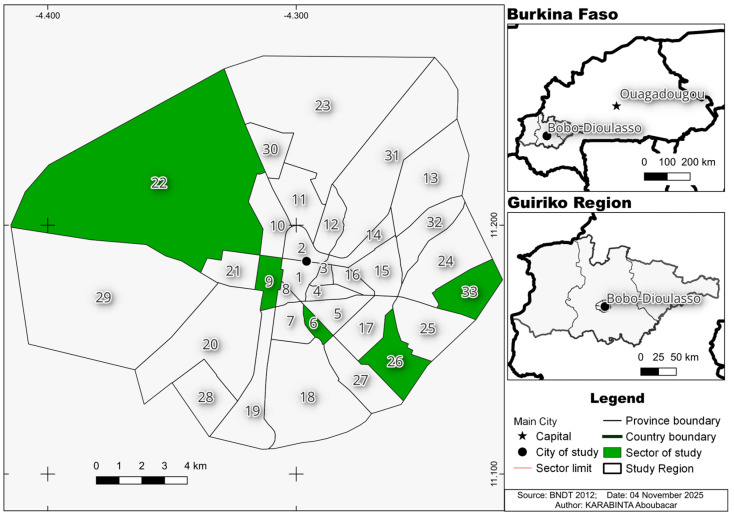
Study sites.

This was a repeated cross-sectional observational study with descriptive and analytical objectives conducted over one year from August 2022 to June 2023. The study was conducted in Bobo-Dioulasso (11°11′00″ N, 4°17′00″ W), located in the western Houet province of Burkina Faso, with a population exceeding 984,603 inhabitants [34]. Bobo-Dioulasso is a cosmopolitan hub linking Côte d’Ivoire, Mali, and Burkina Faso. Five neighborhoods were selected, comprising four urban sectors and one rural site (Figure 1: Study Area). Our choice of study sites is generally based on areas with high human concentration at entry points and on areas surrounded by waste and stagnant water courses. The concessions are taken randomly in the surrounding area.

Sector 9: Situated at the city center, this neighborhood hosts the Bobo-Dioulasso railway station (SITARAIL). The surrounding residential area features a notably disorganized accumulation of railway company materials, which serve as potential *Aedes* breeding sites. Sector 22: This sector encompasses the National Culture Week (Semaine Nationale de la Culture, SNC) venue, attracting tourists biennially from across Africa and other continents, making it a potential hotspot for viral circulation and thus a critical surveillance area. Sector 26: A newly inhabited area characterized by intense anthropogenic activity leading to ecological transformations that likely influence vector distribution. Sector 6: Traversed by the Houet stream, this area suffers from waste dumping and wastewater runoff, creating ideal conditions for vector proliferation.

The urban vector *Aedes aegypti* breeds year-round primarily in clean domestic water containers (including jars, flower pots, water storage basins) and peridomestic sites (including various containers, tires, anthropogenic waste). Its density peaks during the rainy season and in densely populated traditional central city neighborhoods [6,35].

The rural site corresponds to the “Premier Campement,” located approximately 5 km from Bobo-Dioulasso along the Bobo-Dioulasso-Ouagadougou axis (see Figure 1). In this rural environment, other crepuscular or nocturnal *Aedes* species with lower anthropophily develop in natural breeding sites predominantly during the rainy season. These include potential yellow fever vectors such as *Aedes luteocephalus* and *Aedes vittatus*, as well as nuisance species like *Culex tarsalis*, *Aedes hirsutus*, *Aedes fowleri*, and *Aedes argenteopunctatus*.

### 2.2. Sample Collection

Given the large number of potential emerging viruses and the limited availability of very expensive technical resources, it is very difficult to study all of them. Therefore, we focused only on the most emerging diseases and those causing the most epidemics in our subregion. Given that this is a vast field. Our focus in this experiment was on arboviruses vectored by *Aedes* spp. (ZIKV, DENV, CHIKV, and YFV). The collection of *Culex* and *Anopheles* mosquitoes was incidental and indicates that *Aedes* mosquitoes may compete with these genera for ecological resources.

To achieve an acceptable rate of arboviral infection, sampling must be maximized by deploying traps in strategic locations, using specific attractants, and ensuring they are well placed and maintained. Surveillance methods included oviposition traps to collect eggs, larval surveys in some concessions per sector conducted, and adult mosquito collections using BG-Sentinel traps and Prokopack aspirators. It is important to target larval habitats (wet areas, stagnant water) and adult resting places (shaded, vegetated areas), and to place active traps out of reach of curious onlookers. The traps are regularly monitored and maintained. The samples are handled, transported to the Muraz Center laboratory, and stored carefully under normal conditions.

#### 2.2.1. Collection of *Aedes* Eggs by Ovitrap

**Figure 2:**
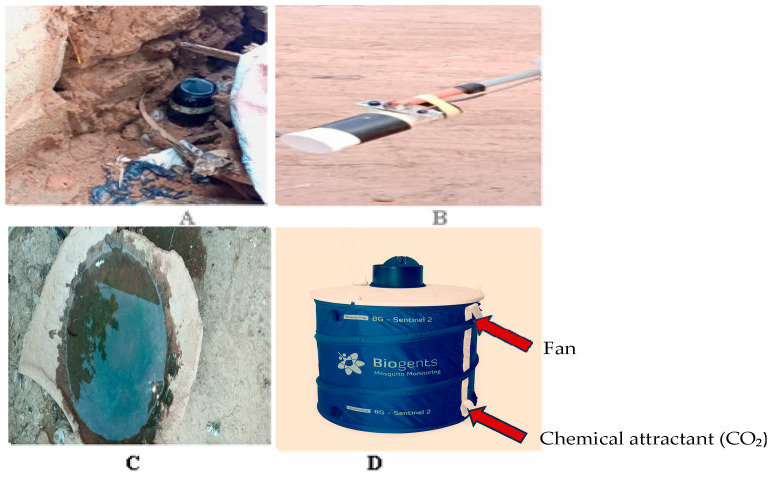
Mosquitos traps and domestic Larval Habitats Identified Through Household Survey. (**A**): Oviposition traps were deployed to attract gravid *Aedes* female mosquitoes seeking sites for egg-laying. Each trap consisted of a black container filled with water, designed to create a visual contrast and simulate favorable breeding conditions. (**B**): The Prokopack is an active, manual device used to collect resting mosquitoes both indoors and outdoors. Unlike the BG-Sentinel trap, this aspirator does not rely on chemical attractants. It functions through a motorized suction mechanism operated by trained field technicians, allowing the capture of mosquitoes in natural resting sites such as walls, ceilings, furniture, and outdoor vegetation. This approach enables the collection of specimens from multiple genera and species, across all gonotrophic stages, thereby providing a comprehensive representation of local vector populations and their resting behavior. (**C**): larva collection in domestic container. (**D**): The BG-Sentinel trap is a passive device specifically designed to attract and capture host-seeking female mosquitoes. Its operation relies on the continuous release of chemical attractants that mimic human skin odor, combined with a carbon dioxide (CO_2_) source that replicates host respiration cues. An integrated fan then draws the attracted mosquitoes into a collection net, where they are retained alive.

The ovitrap is a device designed to attract female *Aedes* mosquitoes and prevent them from laying eggs in natural breeding sites. This trap contains a filter paper substrate on which the females lay their eggs. In total, 30 ovitraps were placed in houses, monthly by sector. As a larval control tool, it effectively reduces Aedes mosquito populations around human dwellings without negatively impacting the surrounding biodiversity. The ovitrap is simple and environmentally friendly, attracting mosquitoes through its black color and the presence of stagnant water. It operates passively, requiring no electricity or chemical attractants (Figure 2A).

#### 2.2.2. Collection of *Aedes* spp. Larvae

Every two months, thirty (30) households per neighborhood were surveyed. All internal and external water-holding containers within each household were inspected for larvae according to the Breteau Index protocol. Larvae collected from each household were grouped separately into indoor and outdoor samples. A bimonthly larval collection was conducted. A total of one hundred and twenty (120) houses were surveyed across four urban neighborhoods of Bobo-Dioulasso, with 30 houses per neighborhood. The rural site was surveyed following the same protocol, with 30 houses sampled, representing a comparable unit to an urban neighborhood in Bobo-Dioulasso (Figure 2C).

#### 2.2.3. Adult Mosquito Collection Using BG-Sentinel Traps

BG-Sentinel traps were deployed monthly using different types of baits, including the “Human Skin Lure” and/or traditional attractants, to capture adult mosquitoes. The traps were set up across five sites (Sectors 6, 9, 22, 26, and 33) within the city of Bobo-Dioulasso. In each sector, four households were selected, with one house per concession. Two BG traps were placed per household, one indoors and one outdoors, resulting in 8 traps per sector (4 households × 2 traps), totaling 16 traps across the two urban sites. Mosquito collections were conducted every 3 h over a continuous 24-h period for 3 consecutive days each month in each sector. The rural site, “Premier Campement de Bobo-Dioulasso,” was treated as a single sector, where eight BG traps were deployed along a transect similar to that used in the urban areas. Collected data allowed for characterization of the vector’s bioecology, including species diversity, breeding sites, and behavior. Blood-fed females were preserved individually in tubes for subsequent analysis of host feeding preferences (Figure 2D).

#### 2.2.4. Collection of Resting Adult Mosquitoes Using Prokopack Aspirators

Resting mosquitoes were collected both indoors and outdoors within bedrooms by two technicians using battery-powered Prokopack aspirators. Aspirations were conducted monthly in thirty (30) households per neighborhood. Each aspiration session, indoors and outdoors per house, lasted 30 min. Collections were performed over three consecutive days, sampling 10 households per day per neighborhood. Strict adherence to the aspiration duration was ensured, with equal sampling time maintained for both indoor and outdoor collections by the technicians. Mosquitoes were caged and transported to the Centre MURAZ Entomology Laboratory for identification (Figure 2B).

### 2.3. Mosquito Identification and Sample Preparation

Mosquitoes from Egg Collection, pupae, adult mosquitoes from BG-Sentinel traps, and prokopack aspirators were first anesthetized by incubation at −20 °C for 5 min, and mosquitoes were identified using a magnifying glass (Leica, S6E, Denaher, Heerbrugg, Switzerland) based on their morphological characteristics. *Aedes aegypti* were confirmed by PCR. All mosquito samples were stored in 1.5 mL microcentrifuge tubes in pools of up to 50 mosquitoes, according to genus, species, sex, and collection sites. Samples were preserved at −80 °C before nucleic acid extraction and arbovirus molecular screening at the Burkina Faso National Reference Laboratory for Viral Haemorrhagic Fevers, Centre MURAZ.

### 2.4. Molecular Screening for Arbovirus Detection

#### 2.4.1. Viral RNA Extraction from Mosquitoes

The mosquito pools were transferred to 2 mL Eppendorf tubes containing phosphate-buffered saline (PBS) (Sigma-Aldrich, Merck KGaA, Darmstadt, Germany) and automatically homogenized using the TissueLyser II (Qiagen, Hilden, Germany 2013–25, LT). The homogenate was then centrifuged at 10,000 rpm at 4 °C for 20 min. Viral RNA was extracted from the supernatant using the QIAamp Viral RNA Mini Kit (Qiagen, Hilden, Germany). Then, RNA was stored at −80 °C until molecular screening.

#### 2.4.2. Real-Time RT-PCR Analysis

DENV, CHIKV, YFV, and ZIKV were screened by real-time RT-PCR using Lyophilized one-step RT-PCR Polymerase Mix (TIB MOLBIOL, Berlin, Germany). The reaction mixture consists of 5 µL of extracted RNA, 10 μL of enzyme, 1 μL (10 μM) of each virus forward primer, 1 μL (10 μM) of each virus reverse primer, 0.5 μL (10 μM) of each virus probe, and 2.5 µL of water, PCR grade. Real-time RT-PCR was performed using MIC PCR Bio molecular Systems (bms, Unit 5/3 Northward Street, Springfield Queenslan, Australia). The thermal cycling profile of this assay consists of a 10-min reverse transcriptase (RT) step at 55 °C, 95 °C for 1 min, and then 40 cycles of 95 °C for 15 s and 60 °C for 30 s. The results were positive if the cycle threshold (Ct) values were equal to or less than 37 cycles. The primers and probe used in this study are shown in Table 1.

### 2.5. Data Management

Field data were collected using electronic tablets and data collection sheets on the Kobo collection. All data was sent to a server. The results of the qRT-PCR analyses were generated using MIC PCR Bio molecular Systems software version 2.8.13 (S/N M0005072 and S/N M000357). All data were recorded using Microsoft Office Excel 2016 software. R software version 4.3.1 was used to analyze the different frequencies. The minimum infection rate (MIR) with a 95% confidence interval was determined using RStudio software version 4.3.1. The MIR assumes that a positive pool contains only one infected mosquito. Generated by micPCR version 2.8.13 at 04/10/2024 19/05/17 LNR/Muraz Bobo-Dioulasso. The minimum infection rate (MIR) is a basic statistical measure used to estimate the prevalence of a pathogen (like a virus) in a population of vectors, such as mosquitoes. MIR assumes that a positive pool contains only one infected mosquito, thereby generally underestimating the true infection rate, especially when prevalence is high.

## 3. Results

### 3.1. Longitudinal Monitoring of Egg Density Collected in Trap Nests Placed on Study Sites

**Figure 3:**
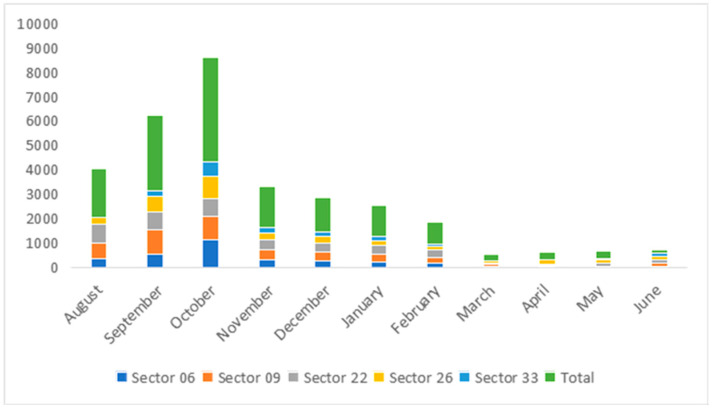
Eggs collected in trap nests by sector and by month from August 2022 to June 2023.

From August 2022 to June 2023, with a peak in October. This curve falls from November, remains constant, and then rises again from June. A total of 16,318 eggs were collected with an average of 3264 eggs per sector (Pearson’s Chi-squared test: χ^2^ = 658.1169; Df = 36; *Pr*(*>Chisq*) < 2 × 10^−16^), resulting in (49%) females and (51%) males. However; urban sectors 9 (Estimate = 1.13243; *Z*-value = 4.479; *Pr*(*>|z|*) < 7.49 × 10^−6^) and 22 (Estimate = 1.58309; *Z*-value = 6.292; *Pr*(*>|z|*) < 3.13 × 10^−10^) are in the majority; and rural sector 33 (Estimate = −0.84338; *Z*-value = −2.566; *Pr*(*>|z|*) = 0.011) is in the minority. Figure 3 illustrates the egg density collected by month and site during the period from.

### 3.2. Density of Larvae and Pupae Collections by Breeding Site

The entomological survey of larvae and pupae in various aquatic habitats revealed a predominance of *Aedes* species across all types of breeding sites sampled between August 2022 and June 2023.

Animal drinkers: *Aedes* larvae accounted for approximately 65% of larvae collected (220 specimens) and nearly 57% of pupae (66 specimens), highlighting this site as a significant breeding habitat for *Aedes*. *Culex* species represented about 27% of larvae and 26% of pupae, while *Anopheles* were absent. Mixed populations composed roughly 11–17% of specimens. No significant difference in species distribution among sources (*p*-value = 0.7552), indicating that egg laying was relatively evenly distributed among species, despite the predominance of *Aedes* at the larval and pupal stages.

Domestic waste containers: These sites showed an even higher dominance of *Aedes*, constituting 66% of larvae (1328 specimens) and 100% of pupae (89 specimens), underscoring their critical role as breeding grounds for *Aedes*. *Culex* larvae were also present (28%), whereas *Anopheles* and mixed populations were minimal or absent at the pupae’s stage. The test statistics confirmed a significant difference in the distribution of species between sources (*p*-value < 1 × 10^−5^).

Utensils: Similar trends were observed with *Aedes*, representing about 51% of larvae (3283 specimens) and 64% of pupae (480 specimens). Notably, *Culex* comprised a substantial portion (37% larvae and 28% pupae), while *Anopheles* were rare (<1%), and mixed populations accounted for around 7–11%. The species distribution of pupae also differed significantly between sources (*p*-value < 1 × 10^−5^). Overall, *Aedes* mosquitoes were the predominant genus in all breeding site types, particularly in domestic waste and utensils (Table 2). The statistical analyses confirm that species composition varies significantly according to the type of breeding site, especially at the larval and pupal stages.

### 3.3. Household and Inhabitant Data Collected During Bimonthly Surveys

The study surveyed a total of 1413 households across five sectors of Bobo-Dioulasso between August 2022 and June 2023, accounting for 3753 sleeping spaces (layers) and a population of 402,753 inhabitants. Sector 33 recorded the highest number of inhabitants (105,109), followed by Sector 26 (83,162) and Sector 6 (74,420) (Table 3). These sectors represent densely populated areas where vector control efforts may have a significant public health impact. The number of households surveyed varied by month and sector, with the highest total number of households visited in October (335) and the lowest in June (190). Similarly, the number of inhabitants fluctuated over time, with a general increasing trend from August (33,096 inhabitants) to June (119,025 inhabitants), which may reflect seasonal population movements or survey coverage differences (Table 3). The number of sleeping spaces per household also varied but remained relatively stable, indicating consistent household sizes during the survey period. These demographic data provide essential context for interpreting entomological findings and planning targeted interventions in areas with high human–mosquito contact risk.

### 3.4. Distribution of Mosquito Species Captured by BG Traps from August 2022 to June 2023

A total of 23,229 mosquitoes were captured using BG-Sentinel traps over the study period, with clear dominance of *Aedes aegypti* (873 females and 386 males) and *Culex* species.

**Figure 4:**
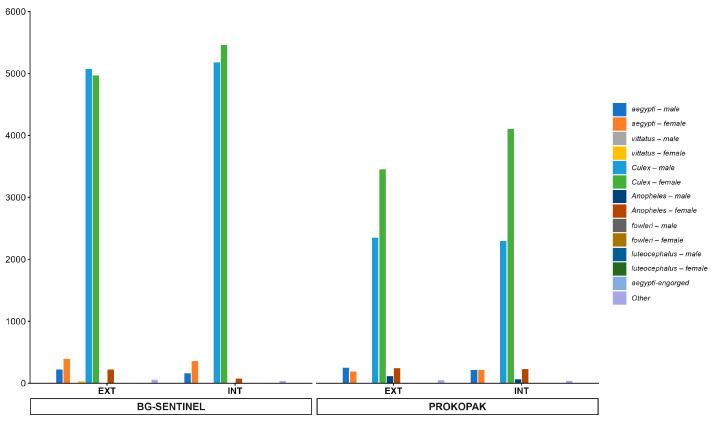
Adult mosquitoes captured according to the capture method and location.

For *Aedes aegypti*, a total of 383 males were collected, with the highest captures in August (187) and February (35). Females numbered 753, predominantly collected in August (401 *Aedes aegypti* females) and October (176 *Aedes aegypti* females) (Figure 4). Females gorged were 60 and 124 pregnant females, indicating ongoing reproductive activity. *Aedes vittatus*: a few specimens were captured (33 females, 2 males), mostly in August, suggesting a limited presence in the study area (sectors 6 and 26). *Aedes Luteocephalus*: very few specimens were captured (3 females, 1 male), mostly in August, suggesting a limited presence in the study area (Figure 4).

The *Culex* species was the most abundant genus captured, with 10,246 males and 10,425 females recorded. Their high numbers were relatively consistent across months, with peaks in August for males, and October for females (Figure 4).

The *Anopheles* species were rarely captured (9 males and 292 females), mostly between August and October, consistent with their lower abundance in urban settings. Other species, such as *Fowleri* and the subgenus *Aedes*, were not captured during the survey (Figure 3).

There is no statistically significant difference between the outdoor and indoor groups for the measured values (*F*-value = 0.942; Df = 1; *Pr*(>*F*) = 0.355).

### 3.5. Distribution of Mosquito Species Prokopak Traps from August 2022 to June 2023

A total of 13,713 mosquitoes, including 1199 *Aedes* mosquitoes (462 males and 737 females), were captured using Prokopak traps over the study period, with clear dominance of *Aedes aegypti* and *Culex* species.

For *Aedes aegypti*, a total of 458 males were collected, with the highest captures in August (406) and February (52). Females numbered 441, predominantly collected in August (406 *Aedes aegypti* females) and October (35 *Aedes aegypti* females). Pregnant females were 205, with some gorged females (81 specimens), indicating ongoing reproductive activity. *Aedes vittatus*: a few specimens were captured (6 females, 2 males), mostly in August, suggesting a limited presence in the study area (sector 6 and sector 26) (Figure 4).

For *Aedes Luteocephalus*, very few specimens were captured (4 females, 2 males), mostly in August, suggesting a limited presence in the study area (Figure 4).

For *Culex*, the most abundant genus captured, with 8199 males and 11,486 females recorded. Capture numbers were high and relatively consistent across months, with peaks in September and December for males, and August and October for females (Figure 4).

For the *Anopheles* species, they were rarely captured (99 males and 202 females), mostly in August, October, and November, consistent with their lower abundance in urban settings. Other species, such as *Fowleri* or other subgenre *Aedes,* were not captured during the survey (Figure 4).

Regardless of the type of trap used (BG-Sentinel or Prokopak), no statistically significant difference was observed between the outdoor and indoor groups for the measured values (*F*-value = 0.942; Df = 1; *Pr*(>*F*) = 0.355). The means of the two groups can therefore be considered similar in this context.

### 3.6. Distribution of Captured Aedes Mosquitoes

A random sample of 9952 mosquitoes of all stages, species, and sexes, collected from all study areas, were tested by RT-PCR testing. This included 401 mosquitoes collected by Prokopak suction capture, 753 mosquitoes collected by BG traps, and 6479 hatched eggs and 2319 emerging mosquitoes. After identification, PCR virus detection tests were performed on all samples. The distribution of these mosquitoes is shown in Table 4. *Aedes* mosquitoes were divided into *n* groups, with a maximum of 50 mosquitoes per group, according to species, stage, sex, type, and collection location. That in an average of 199 pools (9952/50) (Table 4).

The results showed a highly significant effect of trap type on mosquito captures (*F*-value = 29.89; Df = 3; *Pr*(*>F*) < 0.001). Trap nests captured significantly more mosquitoes than all other methods: Trap nests vs. BG trap (Tukey’s post hoc test: *diff* = 1111.6; 95% CI [710.716–1512.483], *p*-value < 0.001); Trap nests vs. Prokopak (*diff* = 1182.0; [781.116–1582.883], *p*-value < 0.001); Trap nests vs. Prospection (*diff* = 798.4; [397.516–1199.283], *p*-value < 0.001). Other pairwise comparisons (Prokopak vs. BG trap, Prospection vs. BG trap, Prospection vs. Prokopak) were not statistically significant (*p*-value > 0.05). These results indicate that Trap nests captured significantly more mosquitoes than all other methods, while captures with Prokopak, BG-Sentinel, and Prospection were comparable.

### 3.7. Molecular Detection of Arboviruses in the Different Study Sites

**Figure 5:**
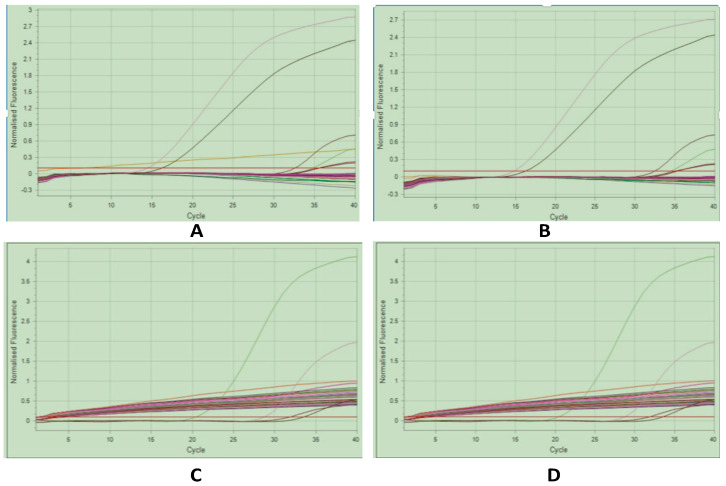
Cycling CHIKV, DENV, and YFV. (**A**) CHIKV sector 9; (**B**) CHIKV sector 26; (**C**) DENV sector 22; (**D**) YFV sector 22.

Overall, all RNA from the 199 pools was screened by RT-PCR for the detection of DENV, ZIKV, YFV, and CHIKV. RT-PCR. The result for the rural sector is negative. The result for the year 2022 is also negative. However, three urban sectors are positive. Regarding the adults emerging from egg-laying and larval prospecting, no virus was detected. No ZIKV was detected. DENV was detected in 2/3 pools of 50 mosquitoes from BG in sector 22, YFV was detected in 1/3 pools of 50 mosquitoes from BG in sector 22, and CHIKV was detected in 1/2 pools of 50 mosquitoes (pregnant and gorged females) taken by aspiration in sectors 9 and in 1/2 pools of 50 mosquitoes in sector 26 taken by BG for the year 2023. The minimum infection rate (MIR) is globaly 24.70%, 95% CI [23.852–25.547] (Table 5) (Figure 5).

## 4. Discussion

This study confirms the predominance of *Aedes aegypti* as the principal vector of the arboviruses tested (dengue and chikungunya) in the urban environment of Bobo-Dioulasso, consistent with previous findings in Burkina Faso and other tropical African regions [40,41]. Our results are consistent with some cases reported in 2023 in Burkina Faso, notably the presence of DENV and CHICV in human serum, as in neighboring Senegal [42]. The minimum infection rate (MIR) is average and significant. The high abundance of *Ae. aegypti* larvae and adults in domestic and peri-domestic habitats, particularly artificial containers, water storage vessels, and household waste, underscores the significant role of human activity in shaping vector ecology. Overall, *Aedes* mosquitoes were the predominant genus in all breeding site types, particularly in domestic waste and utensils, which may serve as key targets for vector control efforts [43]. The absence or very low presence of *Anopheles* larvae and pupae in these breeding habitats aligns with their known preference for different aquatic environments.

Frequently, *Anopheles* larvae and pupae were in mixed breeding sites with *Aedes* and *Culex* [12,44]. This explains the selective nature of breeding sites for each species. This is the same case for female *Aedes* mosquitoes, which prefer laying their eggs in a specific nest box [45,46]. The considerable presence of Culex species likely reflects their involvement in the transmission of other arboviruses, although their role in dengue, chikungunya, and Zika transmission appears secondary in this context. Not forgetting their role in the culicidae nuisance and also for the environmental biotope [47,48]. The presence of the species *luteocephalus* on the periphery confirms its sylvatic status and, therefore, its preference for forested areas. The sparse detection of *Aedes vittatus* and the near absence of *Anopheles* in urban sites suggest a limited contribution of these species to arbovirus transmission in the city, although their presence in rural settings warrants further investigation. Our longitudinal surveillance revealed pronounced seasonal fluctuations, with vector abundance peaking during the rainy months of September and October [49,50]. This aligns with the seasonal availability of stagnant water, facilitating larval development in natural and artificial breeding sites [51]. Notably, urban sectors 9 and 22 exhibited significantly higher larval site density and adult mosquito populations, correlating with dense human habitation and anthropogenic environmental changes. These spatial heterogeneities highlight the need for geographically targeted vector control measures. The predominance of artificial containers as larval habitats further emphasizes the critical importance of environmental management and community engagement in vector control efforts. These results demonstrate the predominance of *Aedes aegypti* and *Culex* mosquitoes in the urban environment of Bobo-Dioulasso, with important implications for arbovirus transmission risk and vector control strategies [52,53]. The integrated use of BG-Sentinel and Prokopack traps enabled comprehensive monitoring of adult mosquito populations, revealing a majority of *Ae. aegypti* females, including gravid individuals, indicate active reproduction and potential for virus transmission. Molecular detection via real-time RT-PCR confirmed the presence of dengue, chikungunya, and yellow fever viruses in mosquito pools, despite the absence of concurrent confirmed human cases [29,54]. This finding suggests cryptic viral circulation within the vector population, reinforcing the indispensable role of entomological surveillance as an early warning system to preempt outbreaks and guide timely public health responses. The study’s outcomes validate an integrated surveillance approach combining larval and adult monitoring with molecular diagnostics to capture a holistic picture of vector dynamics and arbovirus risk. The novel surveillance platform developed facilitates real-time data integration and analysis, supporting prompt, evidence-based decision-making [33,55].

Observed spatial and temporal variations in vector density necessitate adaptive control strategies tailored to high-risk sectors and peak transmission periods [51]. Furthermore, the identification of domestic and peri-domestic artificial breeding sites underscores the importance of the robust entomological data, limitations include restricted rural sampling coverage where secondary vectors may play a greater role, and the lack of contemporaneous human epidemiological data to directly correlate vector indices with disease incidence.

The high population density of *Aedes* recorded, specifically at the egg-laying stage, along with the positive PCR result, would be a precursor sign of the dengue epidemic in 2023. Behind, chikungunya ravages silently. In the study, the absence of viral detection in pools of eggs and larvae was noted, even though they could not determine vertical transmission, which can maintain arboviral circulation in vector populations [30]. This aspect likely involves the stats of mosquito diapause and arboviral diseases, which would cause outbreaks in the dry season. The absence of viral detection in some mosquito pools could neither reflect low infection rates nor the sensitivity thresholds of the molecular assays employed, nor again a sample conservation failure. The rural sites look like sylvan areas. There, mosquito traps can specifically detect sylvatic arboviral circulation.

DENV, CHIKV, YFV, and Zika are the major arboviruses responsible for human infection and are transmitted in sylvatic cycles involving vertebrate animals and wild mosquitoes [56]. Also, reverse transmission is possible, causing outbreaks and hindering arbovirus eradication. Arbovirus emergence or re-emergence poses an imminent public health threat; consequently, careful surveillance of arboviruses in the human population and the sylvatic environment is crucial to implement appropriate public health measures to prevent outbreaks [40]. Globally, the emergence or re-emergence of arboviruses in a public health emergency is considered a notifiable disease. The detection of cases at a given site is sufficient to declare an epidemic [29]. Expanding geographic scope, integrating clinical surveillance, and establishing systematic insecticide resistance monitoring are critical next steps to refine vector control policies. In the same context, a brigade “laabal” against insalubrity and against all forms of incivility was born in Burkina Faso, it is responsible for surveillance, unblocking of gutters, drainage of soils, elimination of shelters under penalty of fine to the residents in case of discovery of garbage of stagnant puddles of used water favoring the express creation of shelter by discharging waste water in the streets or blocking the gutters. Thus, development through hygiene must begin with oneself. Ongoing evaluation and optimization of the surveillance platform will further enhance its utility for sustainable arbovirus risk management in Burkina Faso and comparable urban settings across West Africa.

## 5. Conclusions

Based on the PCR results of our research on mosquito virus detection, we identified CHIKV and DENV at a site plagued by recurrent dengue epidemics. The results confirm some findings recorded in human cases in Burkina Faso. This study highlights the critical role of an integrated entomological surveillance platform in understanding and managing arboviral disease risks in Bobo-Dioulasso, Burkina Faso. The predominance of *Aedes aegypti* in diverse urban breeding sites, coupled with the detection of dengue, chikungunya, and yellow fever viruses in mosquito populations, underscores the persistent threat posed by arboviral diseases in the region. Seasonal fluctuations in vector abundance emphasize the need for timely, context-specific vector control strategies. The innovative surveillance system established here not only enhances real-time monitoring and data-driven decision-making but also provides a scalable framework for arboviral epidemic preparedness across similar urban settings in West Africa. Strengthening such platforms, alongside continuous entomological and virological monitoring, is essential to mitigate future outbreaks and protect public health. Our study, being a pilot project, and given its objectives and resources, has limitations, namely the exclusion of other arboviruses such as West Nile Virus (WNV) and Rift Valley fever virus (RVFV). This aspect can be addressed in our upcoming research and within a broader context.

## Figures and Tables

**Table 1:** Primers and probes used in this study.

Virus	Primer/Probe	Sequence (5′ to 3′)	Reference
DENV	Forward	AAGGACTAGAGGTTAKAGGAGACCC	[36]
	Reverse	GGCCYTCTGTGCCTGGAWTGATG	
	Probe	5′-6FAM-AACAGCATATTGACGCTGGGARAGACC-BBQ	
CHIKV	Forward	AAG CTY CGC GTC CTT TAC CAA G	[37]
	Reverse	CCA AAT TGT CCY GGT CTT CCT	
	Probe	6′-FAM-CCA ATG TCY TCM GCC TGG ACA CCT TT-TMR	
ZIKV	Forward	AARTACACATACCARAACAAAGTGGT	[38]
	Reverse	TCCRCTCCCYCTYTGGTCTTG	
	Probe	5′-FAM-CTYAGACCA+G+C+T+GAAR--BBQ	
YFV	Forward	ATTGAGGTGYATTGGTCTGC	[39]
	Reverse	GTCRRTTCTCTGCTAATCGCTCA	
	Probe	5′-6FAM-AGTTGCTAGGC+A+AT+A+A+A--BBQ	

**Table 2:** Larva’s breeding sites surveyed from August 2022 to June 2023.

Labels Lines	Eggs	Larvae	Pupae	*p*-Value
Animal drinker				0.7552
*Aedes*	64.71% (11)	58.36% (220)	56.9% (66)	
*Culex*	23.53% (4)	27.59% (104)	25.86% (30)	
*Anopheles*	0% (0)	0% (0)	0% (0)	
Mixed	11.76% (2)	14.06% (53)	17.24% (20)	
Total	100% (17)	100% (377)	100% (116)	
Domestic waste				<1 × 10^−5^
*Aedes*	66.1% (39)	61.34% (1328)	100% (89)	
*Culex*	23.73% (14)	28.04% (607)	0% (0)	
*Anopheles*	3.39% (2)	1.39% (30)	0% (0)	
Mixed	6.78% (4)	9.24% (200)	0% (0)	
Total	100% (59)	100% (2165)	100% (89)	
Utensil				<1 × 10^−5^
*Aedes*	70.59% (96)	51.27% (3283)	64.17% (480)	
*Culex*	21.32% (29)	36.62% (2345)	27.54% (206)	
*Anopheles*	0.74% (1)	0.78% (50)	0% (0)	
Mixed	7.35% (10)	11.32% (725)	8.29% (62)	
Total	100% (136)	100% (6403)	100% (748)	

**Table 3:** Number of inhabitants registered per household, month, and per sector during the bimonthly survey.

Labels Lines	August	October	December	February	April	June	Total
Sector 6							
No. household	85	131	88	52	57	45	458
layer	268	240	247	135	168	201	1259
Number of people	1248	5805	10,335	15,196	18,031	23,805	74,420
Sector 9							
No. household		99	65	47	40	51	302
layer		170	131	119	106	125	651
Number of people	229	3996	8265	13,935	18,447	22,782	67,654
Sector 22							
No. household		35	32	35	32	34	168
layer		106	69	84	98	90	447
Number of people		4905	9435	14,835	21,105	22,128	72,408
Sector 33							
No. household	90	32	59	41	16	30	268
layer	233	154	175	101	38	64	765
Number of people	29,294	7505	20,790	13,035	8880	25,605	105,109
Sector 26	0	0	0				
No. household	30	38	46	32	41	30	217
layer	132	102	102	104	108	83	631
Number of people	2325	6885	11,235	17,174	20,838	24,705	83,162
Total No. household	205	335	290	207	186	190	1413
Total No. layer	633	772	724	543	518	563	3753
Total No. of people	33,096	29,096	60,060	74,175	87,301	119,025	402,753

**Table 4:** Distribution of *Aedes* mosquitoes tested by RT-PCR.

Labels Lines	Sector 6	Sector 9	Sector 22	Sector 26	Sector 33	Total
**BG Trap**	133 (18%)	188 (25%)	234 (31%)	131 (17%)	67 (09%)	753 (100%)
**Prokopak**	13 (04%)	222 (55%)	38 (09%)	71 (18%)	57 (14%)	401 (100%)
**Trap nests**	957 (16%)	1684 (26%)	1619 (25%)	1231 (20%)	820 (13%)	6311 (100%)
**Prospection**	354 (15%)	505 (22%)	760 (33%)	450 (19%)	250 (11%)	2319 (100%)
**Total**						9952 (100%)

**Table 5:** Mosquitoes distribution by area and pool tested results.

Sector	N/S Mosquitoes	Collection Date	Pool/50 of 2022	CHIKV+	DENV+	YFV+	ZIKV+	Collection Date	Pool/50 of 2023	CHIKV+	DENV+	YFV+	ZIKV+	Ct
**6 (urban)**	Adult (133)	Sept–Nov	1 male, 1 female	0	0	0	0	April–June	1 female	0	0	0	0	>37
	Adult (13)		7 Aedes male	0	0	0	0		6 Aedes female	0	0	0	0	>37
	Egg (957)		6 male, 10 female	0	0	0	0		1 male, 2 female	0	0	0	0	>37
	Larvae (354)		2 male, 3 female	0	0	0	0		1 male, 1 female	0	0	0	0	>37
**9 (urban)**	Adult (188)	Sept–Nov	1 male, 1 female	0	0	0	0	April–June	1 male, 1 female	0	0	0	0	>37
	Adult (222)		1 male, 2 female	0	0	0	0		1 male, 1 female	1/2	0	0	0	35
	Egg (1684)		11 male, 18 female	0	0	0	0		1 male, 3 female	0	0	0	0	>37
	Larvae (505)		3 male, 4 female	0	0	0	0		1 male, 1 female	0	0	0	0	>37
**22 (urban)**	Adult (234)	Sept–Nov	1 male, 1 female	0	0	0	0	April–June	1 male, 2 female	0	2/3	1/3	0	31-31-33
	Adult (38)		20 Aedes female	0	0	0	0		18 Aedes male	0	0	0	0	>37
	Egg (1619)		9 male, 20 female	0	0	0	0		1 male, 2 female	0	0	0	0	>37
	Larvae (760)		5 male, 7 female	0	0	0	0		1 male, 2 female	0	0	0	0	>37
**26 (urban)**	Adult (131)	Sept–Nov	1 male	0	0	0	0	April–June	2 female	1/2	0	0	0	32
	Adult (71)		1 female	0	0	0	0		1 male	0	0	0	0	>37
	Egg (1231)		7 male, 15 female	0	0	0	0		1 male, 2 female	0	0	0	0	>37
	Larvae (450)		2 male, 5 female	0	0	0	0		1 male, 1 female	0	0	0	0	>37
**33 (rural)**	Adult (67)	Sept–Nov	1 male	0	0	0	0	April–June	1 female	0	0	0	0	>37
	Adult (57)		7 Aedes female	0	0	0	0		1 male	0	0	0	0	>37
	Egg (820)		5 male, 9 female	0	0	0	0		1 male, 2 female	0	0	0	0	>37
	Larvae (250)		1 male, 2 female	0	0	0	0		1 male, 1 female	0	0	0	0	>37
**Pool (m)**	**199 Pools (9952)**		**159 Pools (7928 m)**						**42 Pools (2024 m)**					
**MIR (%) [95% CI]**			**0%**						**24.7%; 95% CI [** **23.852** **–** **25.547** **]**					

Pool (m): Pool (mosquito); N/S mosquitoes: Number of mosquitoes per stage.

## Data Availability

The original contributions presented in this study are included in the article. Further inquiries can be directed to the corresponding author.
